# *PDL1* expression in inflammatory breast cancer is frequent and predicts for the pathological response to chemotherapy

**DOI:** 10.18632/oncotarget.3642

**Published:** 2015-04-11

**Authors:** François Bertucci, Pascal Finetti, Cécile Colpaert, Emilie Mamessier, Maxime Parizel, Luc Dirix, Patrice Viens, Daniel Birnbaum, Steven van Laere

**Affiliations:** ^1^ Département d'Oncologie Moléculaire, “Equipe Labellisée Ligue Contre le Cancer”, Centre de Recherche en Cancérologie de Marseille (CRCM), Institut Paoli-Calmettes, INSERM UMR1068, CNRS UMR725, Marseille, France; ^2^ Département d'Oncologie Médicale, CRCM, Institut Paoli-Calmettes, Marseille, France; ^3^ Faculté de Médecine, Aix-Marseille Université, Marseille, France; ^4^ Department of Pathology, GZA Hospitals Sint-Augustinus, Antwerp, Belgium; ^5^ Center for Oncological Research (CORE), Faculty of Medicine and Health Sciences, University of Antwerp, Antwerp Belgium

**Keywords:** chemotherapy, inflammatory breast cancer, immune response, PDL1, survival

## Abstract

We retrospectively analyzed *PDL1* mRNA expression in 306 breast cancer samples, including 112 samples of an aggressive form, inflammatory breast cancer (IBC). *PDL1* expression was heterogeneous, but was higher in IBC than in non-IBC. Compared to normal breast samples, *PDL1* was overexpressed in 38% of IBC. In IBC, *PDL1* overexpression was associated with estrogen receptor-negative status, basal and ERBB2-enriched aggressive subtypes, and clinico-biological signs of anti-tumor T-cell cytotoxic response. *PDL1* overexpression was associated with better pathological response to chemotherapy, independently of histo-clinical variables and predictive gene expression signatures. No correlation was found with metastasis-free and overall specific survivals. In conclusion, *PDL1* overexpression in IBC correlated with better response to chemotherapy. This seemingly counterintuitive correlation between expression of an immunosuppressive molecule and improved therapeutic response may be resolved if *PDL1* expression is viewed as a surrogate marker of a strong antitumor immune response among patients treated with immunogenic chemotherapy. In such patients, PDL1 inhibition could protect activated T-cells or reactivate inhibited T-cells and improve the therapeutic response, notably when associated with immunogenic chemotherapy.

## INTRODUCTION

Inflammatory breast cancer (IBC) is an aggressive form of breast cancer with strong metastatic potential [1, 2]. Nearly 60% of patients die from metastatic relapse despite a multidisciplinary treatment including anthracycline/taxane-based neo-adjuvant chemotherapy (combined with trastuzumab for ERBB2-positive cases), followed by radical surgery and adjuvant radiotherapy. Pathological complete response (pCR) to neo-adjuvant chemotherapy (obtained in 15–30% of cases) is a favorable prognostic feature. Adjuvant systemic therapy includes hormone therapy for estrogen receptor (ER)-positive tumors and trastuzumab in case of ERBB2-positivity [3, 4]. However, the 5-year survival remains inferior to 40%. Even more than in non-IBC, the identification of new therapeutic targets is crucial in IBC, justifying the biological studies published for many decades [5, 6] and recently based on high-throughput molecular analyses [7].

The importance of immunity has emerged in breast cancer more recently than in other cancers. Several immune response-related variables have a favorable predictive impact in terms of survival and response to chemotherapy in non-IBC [8–17]. Immune response is a complex phenomenon balanced between activator and inhibitor pathways. Cancer cells can maintain an immunosuppressive microenvironment that favors tumor progression. Programmed cell death 1 (PD1) receptor-ligand interaction is a major inhibitor pathway. Programmed death-ligand 1 (PDL1 or CD274), one of the ligands of PD1, is expressed at the surface of many cancer and immune cells such as antigen-presenting cells. Its binding to PD1 suppresses T-cell migration, proliferation and secretion of cytotoxic mediators, and restricts tumor cell killing [18–24]. PDL1 is upregulated in many different cancers and its blocking enhances anti-cancer immunity. Clinical trials testing anti-PD1 or anti-PDL1 drugs have shown promising results with durable responses in different cancers including melanoma, renal, lung, prostate and bladder carcinomas [25–27]. PDL1 expression by tumor and/or infiltrating immune cells has been shown to correlate with a therapeutic response [25, 26, 28–30].

PDL1 expression has been studied in different cancers [31–42], with evidence of histo-clinical correlations in several studies. A few studies have been reported in non-IBC [43–51], but never in IBC. Here, we have retrospectively analyzed *PDL1* mRNA expression in 112 IBC profiled using DNA microarrays to determine its prevalence and to search for correlations with histo-clinical features, including response to chemotherapy and survival.

## RESULTS

### *PDL1* expression is higher in IBC than non-IBC

We analyzed *PDL1* expression in clinical samples of 112 IBC and 194 non-IBC collected within the World IBC Consortium. Their clinical characteristics are shown in Table 1. IBC patients were younger than non-IBC patients, and IBC samples showed more often than non-IBC samples poor-prognosis features (*p* < 0.01; Fisher's exact test): AJCC stage 3–4, ductal type, high grade, ER-negative, PR-negative and ERBB2-positive status, and aggressive molecular subtypes (basal, ERBB2-enriched). The 5-year metastasis-free survival (MFS) was 49% (95%CI: 37–64%) in IBC patients and 82% (95%CI: 76–88%) in non-IBC patients (*p* = 2.7E-9; log-rank test). Such expected differences confirmed the coherence of our data set.

**Table 1:** Histo-clinical characteristics of IBC and non-IBC samples

Characteristics*	*N*	IBC (*N* = 112)	non-IBC (*N* = 194)	*P*.value
Age, years				**6.67E–03**
<=50	115	53 (48%)	62 (32%)	
> 50	189	57 (52%)	132 (68%)	
AJCC stage				**3.35E–55**
1	65	0 (0%)	65 (34%)	
2	97	0 (0%)	97 (51%)	
3	106	85 (76%)	21 (11%)	
4	34	27 (24%)	7 (4%)	
Histological type				**9.23E–04**
Ductal	250	101 (92%)	149 (77%)	
Other	54	9 (8%)	45 (23%)	
Histological grade				**3.76E–16**
1	50	0 (0%)	50 (26%)	
2	108	27 (25%)	81 (42%)	
3	140	79 (75%)	61 (32%)	
ER status				**2.12E–03**
Negative	97	48 (43%)	49 (25%)	
Positive	209	64 (57%)	145 (75%)	
PR status				**6.58E–05**
Negative	106	55 (49%)	51 (26%)	
Positive	200	57 (51%)	143 (74%)	
ERBB2 status				**4.88E–04**
Negative	248	79 (71%)	169 (87%)	
Positive	58	33 (29%)	25 (13%)	
Molecular subtypes				**1.00E–06**
Basal	57	28 (25%)	29 (15%)	
ERBB2-enriched	47	28 (25%)	19 (10%)	
Luminal A	115	21 (19%)	94 (48%)	
Luminal B	62	22 (20%)	40 (21%)	
Normal-like	25	13 (12%)	12 (6%)	
5-year MFS**	269	49% (CI95 37–64)	82% (CI95 76–88)	**2.66E–09**
Follow-up, median (months)	269	43	75	

* data were missing for some characteristics: age and histological type for 2 cases (<1%), AJCC stage for 4 (<1.5%), and histological grade for 8 (2.6%)

** assessed in the non-stage 4 cases and missed for 1 case

**Figure 1:**
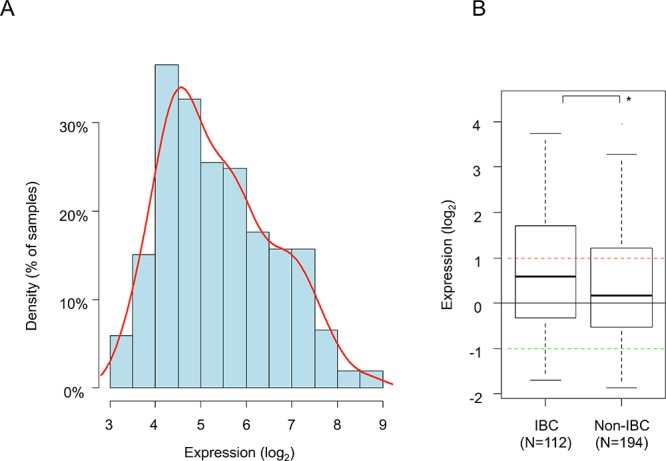
PDL1 mRNA expression across clinical IBC and non-IBC samples **A.** Histogram of distribution of *PDL1* expression levels (log_2_) across the 112 IBC samples (log_2_ scale) after normalization. The red line represents the density curve of distribution. **B.** *PDL1* expression level (log_2_) reported as a box plot according to the type of samples: IBC and non-IBC. The black horizontal line represents the expression level in normal breast samples, the red and green lines represent the thresholds for “PDL1-high” and “PDL1-low” groups respectively. The *p*-value is indicated (Student's *t*-test) are indicated as follows: *, *p* < 0.05.

*PDL1* expression level was heterogeneous across IBC samples with a range of intensities over 3 decades in log_2_ scale (Figure 1A). A similar range of expression was observed in non-IBC samples, but expression was higher in IBC than non-IBC samples (*p* = 0.02, Student's *t*-test; Figure 1B). As compared to normal breast (NB) samples, 38% of IBC samples (42 out of 112) showed *PDL1* overexpression (ratio T/NB ≥ 2; hereafter defined as “PDL1-high” group) and 62% (70 out of 112) did not show overexpression (ratio <2; “PDL1-low” group). For comparison, 28% of non-IBC samples showed *PDL1* overexpression.

### Correlations of *PDL1* expression with histo-clinical characteristics in IBC

We searched for correlations between *PDL1* expression status (high- *versus* low- groups) and histo-clinical variables in IBC samples (Table 2). No correlation was found with patients' age, AJCC stage, histological type and grade, PR and ERBB2 status. By contrast, *PDL1* expression correlated with the ER status and the molecular subtype of samples (Fisher's exact test). Tumors in the “PDL1-high” group were more frequently ER-negative than tumors in the “PDL1-low” group (57% *vs* 34%, *p* = 0.029). The percentages of ERBB2-enriched subtypes and basal subtypes were higher in the “PDL1-high” group than “PDL1-low” group (36 *vs* 19%, and 33 *vs* 20%, respectively), whereas the percentages of luminal A subtypes and luminal B subtypes were lower (10 *vs* 24%, and 10 *vs* 26%, respectively).

**Table 2:** Correlations of *PDL1* expression with histo-clinical characteristics in IBC

Characteristics*	*N*	IBC		*P*.value
		PDL1-low	PDL1-high	
Age, years				0.693
<=50	53	35 (50%)	18 (45%)	
> 50	57	35 (50%)	22 (55%)	
AJCC stage				0.37
3	85	51 (73%)	34 (81%)	
4	27	19 (27%)	8 (19%)	
Histological type				0.151
Ductal	101	62 (89%)	39(98%)	
Other	9	8 (11%)	1(2%)	
Histological grade				0.248
1	0	0 (0%)	0 (0%)	
2	27	20 (30%)	7 (18%)	
3	79	47 (70%)	32 (82%)	
ER status				**2.94E–02**
Negative	48	24 (34%)	24 (57%)	
Positive	64	46 (66%)	18 (43%)	
PR status				0.436
Negative	55	32 (46%)	23 (55%)	
Positive	57	38 (54%)	19 (45%)	
ERBB2 status				0.67
Negative	79	48 (69%)	31 (74%)	
Positive	33	22 (31%)	11 (26%)	
Molecular subtypes				**2.01E–02**
Basal	28	14 (20%)	14 (33%)	
ERBB2-enriched	28	13 (19%)	15 (36%)	
Luminal A	21	17 (24%)	4 (10%)	
Luminal B	22	18 (26%)	4 (10%)	
Normal-like	13	8 (11%)	5 (12%)	

* data were missing for some characteristics: age and histological type for 2 cases (<2%), and histological grade for 6 (5%)

### Correlations of *PDL1* expression with immune parameters

Given the role of PDL1 in immunity, we searched for correlations (Fisher's exact test) between *PDL1* expression and immunity-related factors in IBC samples (Table 3). First, we found a correlation with the lymphocyte infiltrate (both peri-tumoral and intra-tumoral tumor-infiltrative lymphocytes, TILs), available for 44 samples and scored in four categories (none, small, moderate, or strong infiltrate): the percentage of “PDL1-high” samples increased with the degree of lymphocyte infiltrate (*p* = 0.001). Second, *PDL1* expression was associated with T-cell-specific, CD8+ T-cell-specific and B-cell-specific gene expression signatures [52]: the percentage of samples with higher expression of these signatures was higher in the “PDL1-high” group (*p* < 0.01). Third, *PDL1* expression was associated (*p* < 0.0001) with two gene expression signatures (LCK metagene [10] and 28-kinase metagene [11]) reflecting the immune response and in particular cytotoxic T-cell response. Finally, the probability of activation [53] of IFNα, IFNγ, and TNFα pathways was higher in the “PDL1-high” group (*p* < 0.00001; Fisher's exact test). Altogether, these results suggested that *PDL1* expression in IBC is associated with anti-tumor T-cell response.

**Table 3:** Correlations of *PDL1* expression with immune-related parameters in IBC

Characteristics	*N*	IBC		*P*.value
		“PDL1-low”	“PDL1-high”	
**Lymphocyte infiltrate**				**1.54E–03**
0	9	8 (33%)	1 (5%)	
1	13	9 (38%)	4 (20%)	
2	11	6 (25%)	5 (25%)	
3	11	1 (4%)	10 (50%)	
**T-cell metagene**				**1.16E–04**
High	43	17 (24%)	26 (62%)	
Low	69	53 (76%)	16 (38%)	
**CD8+ T-cell metagene**				**1.09E–02**
High	34	15 (21%)	19 (45%)	
Low	78	55 (79%)	23 (55%)	
**B-cell metagene**				**2.74E–06**
High	45	16 (23%)	29 (69%)	
Low	67	54 (77%)	13 (31%)	
**LCK metagene**				**5.11E–05**
High	35	12 (17%)	23 (55%)	
Low	77	58 (83%)	19 (45%)	
**28-kinase metagene**				**2.96E–06**
High	22	4 (6%)	18 (43%)	
Low	90	66 (94%)	24 (57%)	
**IFNα biological pathway**				**6.06E–06**
Not activated	52	44 (63%)	8 (19%)	
Activated	60	26 (37%)	34 (81%)	
**IFNγ biological pathway**				**4.34E–09**
Not activated	53	48 (69%)	5 (12%)	
Activated	59	22 (31%)	37 (88%)	
**TNFα biological pathway**				**4.64E–09**
Not activated	56	50 (71%)	6 (14%)	
Activated	56	20 (29%)	36 (86%)	

### Biological processes associated with *PDL1* overexpression in IBC

Supervised analysis identified 1, 774 genes differentially expressed between the “PDL1-high” group (*N* = 42) and the “PDL1-low” group (*N* = 70), including 1, 607 genes overexpressed and 167 genes underexpressed in the “PDL1-high” group (Supplementary Table 1). Ontology analysis of the 1, 607 genes overexpressed (Supplementary Table 2) revealed a major involvement in the regulation of local immune response, notably the activation of T-cells. Many genes coded for proteins related to T-cell receptor signaling (e.g. *TCR alpha, beta, delta, CD2, CD3D, CD3E, CD8A, CD247, KLRK1*), T-cells differentiation (e.g. *CD27, EOMES, STAT1, STAT4*), T-cells activation (e.g. *ITK, JAK3, LCK, ZAP70*), cytotoxic effector molecules (e.g. *GZMA/B/H/K, C1QA/B, GNLY, PRF1*), inflammation/anti-tumor cytokines (e.g. *IL2RA, IL2RB, IL2RG, IL12RB1, IL12RB2, IL15, IL15RA, IL18BP, IL18, IL21R, IL27RA*, interferon gamma (IFNG) and its receptor, as well as many interferon-induced proteins, *TNF, LTB*), and chemokines related to T-cells activation and homing (e.g. *CCL2/4/5/8/18, CXCL1, CXCL9/10/11*, chemokine (C-C or C-X-C motifs) receptors). In addition, several overexpressed genes were MHC-related molecules, involved in the processing of endogenous antigens and presentation to cytotoxic and helper T-cells: HLA-I or HLA-I-related molecules (e.g. *HLA-A/B/C/E/F/G*, butyrophilin family members), but also HLA-II molecules (e.g. *HLA-DM/DO/DP/DQ/DR, CD74*), and molecules involved in the degradation of cytosolic peptides across the endoplasmic reticulum into the membrane-bound compartment where class I molecules assemble (e.g. *TAP1, TAPBP*, many proteasome subunits). Interestingly, *CTLA4* and *LAG3,* which code for markers of T-cells exhaustion, were strongly overexpressed in the “PDL1-high” group. *HAVCR2* (*TIM3*), another marker of T-cells exhaustion, was also upregulated in the “PDL1-high” group, whereas *PD1* or *BTLA* molecules were not. *PRDM1* (also known as *BLIMP1*); *IDO* and *TGFβ1*, which code for cytokines synthesized in exhausted T-cells, were overexpressed in this group as well. Genes overexpressed in the “PDL1-low” group were involved in the response to hormone stimuli.

### *PDL1* expression correlates with pathological response to chemotherapy in IBC

The pathological response to neo-adjuvant anthracycline-based chemotherapy was documented for 66 out of 112 patients with IBC, of which 22 (33%) had achieved pCR (pCR group) and 44 (67%) had not (no-pCR group). Univariate analysis for pCR prediction (logit link test; Table 4) showed that *PDL1* expression was associated with pCR: the pCR rate was 50% in the “PDL1-high” group *versus* 22% in the “PDL1-low” groups (*p* = 0.03) with an OR for pCR equal to 3.4 (95%CI 1.04–11.51). By contrast, patients' age, histological type and grade, ER, progesterone receptor (PR) and ERBB2 status, and molecular subtypes were not, with a trend for better response in PR-negative *versus* PR-positive tumors (*p* = 0.059). Regarding the two signatures reported as predictive for pathological response in non-IBC, the FAC/T response signature was associated with pathological response in our IBC series (*p* = 0.046) and the Stromal signature tended to be associated (*p* = 0.052). In multivariate analysis incorporating the four variables with a *p*-value inferior to 0.10 in univariate analysis (PDL1 group, PR status, and the two signatures), only the PDL1 group remained significant (*p* = 0.046), suggesting independent predictive value (Table 4).

**Table 4:** Univariate and multivariate analysis for pathological response to neo-adjuvant chemotherapy in IBC

Characteristics	Univariate analysis			Multivariate analysis		
	*N*	OR [CI95]	*p*-value	*N*	OR [CI95]	*p*-value
Age, years > 50 vs. <=50	64	0.72 [0.29–1.72]	0.533			
Histological type, other vs. ductal	64	0.38 [0.04–1.94]	0.392			
Histological grade, 3 vs. 2	60	1.11 [0.42–3.05]	0.859			
ER status, positive vs. negative	66	0.58 [0.24–1.37]	0.296			
PR status, positive vs. negative	66	0.36 [0.14–0.86]	**0.059**	66	0.49 [0.17–1.38]	0.258
ERBB2 status, positive vs. negative	66	0.48 [0.15–1.3]	0.247			
Molecular subtypes, ERBB2-enriched vs. basal	66	0.95 [0.28–3.18]	0.947			
Luminal A vs. basal	66	0.24 [0.05–0.94]	0.110			
Luminal B vs. basal	66	0.67 [0.16–2.55]	0.627			
Normal-like vs. basal	66	0.5 [0.12–1.81]	0.391			
PDL1 group, high vs. Low	66	3.44 [1.42–8.63]	**2.33E–02**	66	3.49 [1.28–10.1]	**4.46E–02**
FAC/T response signature, “pCR-predicted” vs. “no pCR-predicted”	66	3 [1.22–7.54]	**4.60E-02**	66	1.58 [0.53–4.7]	0.487
Stromal signature, “pCR-predicted” vs. “no pCR-predicted”	66	2.86 [1.18–7.08]	**0.052**	66	3.28 [1.25–9.17]	0.047

### Correlations of *PDL1* expression with survival in IBC

**Figure 2:**
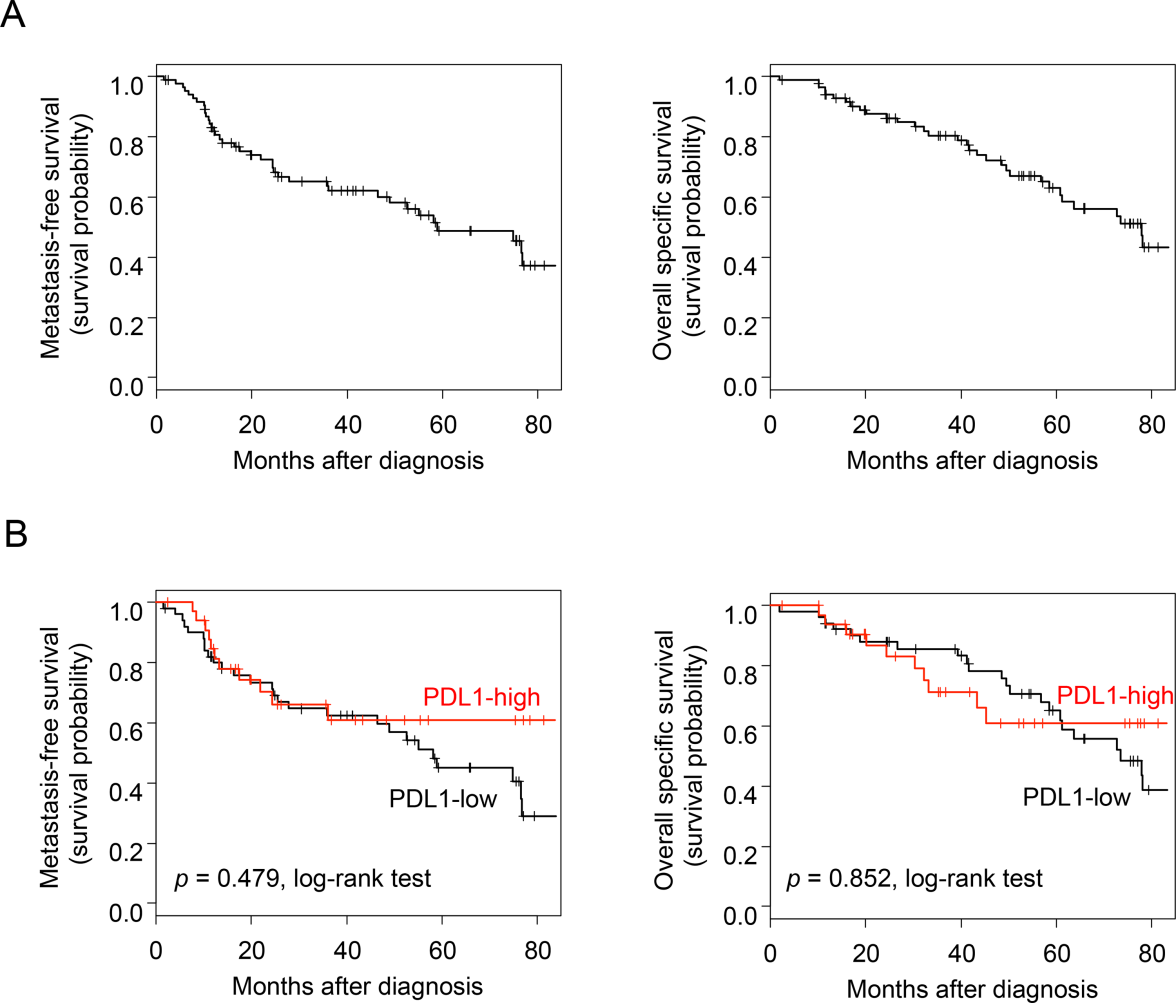
Survival in patients with IBC **A.** Kaplan-Meier MFS (*left*) and OSS (*right*) curves in the 85 patients with IBC. **B.** Similar to (A), but according to the *PDL1* expression status (red curve: “PDL1-high” group *versus* black curve: “PDL1-low” group).

We first assessed the prognostic value of *PDL1* expression in term of MFS, which was annotated for 85 patients with stage 3 IBC: 46 remained metastasis-free during a median follow-up of 43 months (median MFS: 59 months) and 39 displayed metastatic relapse. The 5-year MFS rate was 49% (95%CI: 37–64%) (Figure 2A). In univariate analysis, *PDL1* expression was not associated with MFS (*p* = 0.479, log-rank test; Figure 2B), whereas ERBB2 status was (*p* = 0.046) and ER status tended to be associated with MFS (*p* = 0.075; Supplementary Table 3).

The results were similar with respect to overall specific survival (OSS) for the 85 patients, including 49 who remained alive during a median follow-up of 53 months (median OSS: 78 months) and 36 who died from disease progression. The 5-year OSS was 63% (95%CI: 52–76%) (Figure 2A). *PDL1* expression was not associated with OSS in our IBC series (*p* = 0.852, log-rank test; Figure 2B), whereas ERBB2 status was (*p* = 0.010) and ER and PR statutes tended to be associated with OSS (*p* = 0.057 and *p* = 0.053 respectively; Supplementary Table 3).

## DISCUSSION

IBC in an aggressive form of breast cancer that could benefit from innovating therapeutic strategies. Given the promising results of PDL1 inhibitors in different cancers, we aimed at documenting the expression of *PDL1* in a series of clinical IBC samples and to search for histo-clinical correlations. *PDL1* overexpression was found in more than one third of samples and correlated with aggressive molecular subtypes (basal and ERBB2-enriched) and better pathological response to chemotherapy.

So far, PDL1 expression in cancers has been essentially studied at the protein level using IHC. Here, we based our analysis on mRNA expression measured using DNA microarrays for several reasons. First, PDL1 IHC is not yet standardized and many discordant results have been reported across studies, notably in prognostic studies [54]. Several antibodies are available but lack specificity and reproducibility [55, 56] and the optimal positivity cut-off is not defined. Second, a positive relationship between protein and mRNA PDL1 expression has been reported in non-IBC [46]. Finally, similar results have been reported in non-IBC at the mRNA level with DNA microarrays and *in situ* hybridization (ISH) [46].

We found *PDL1* overexpression in 38% of IBC samples. To date, six teams including ours have described *PDL1* expression in non-IBC [43–49, 51], with different analytic levels (protein, RNA) and different scoring systems, the rate of expression/overexpression reported ranging between 23 and 55%. In a large series of 5, 454 non-IBC samples, we found *PDL1* overexpression in 20% of cases [50]. Here, with the same analytic method for IBC and non-IBC samples, we observed higher expression in IBC than in non-IBC, and the rate of overexpression was higher in IBC (38% *vs* 28%).

*PDL1* expression was heterogeneous in IBC, with a 3-log range of expression levels allowing the search for correlations with other tumor features. “PDL1-high” IBC samples were more frequently ER-negative and more frequently ERBB2-enriched and basal than “PDL1-low” samples. These correlations persisted when *PDL1* expression was analyzed as continuous value (Wilcoxon's test; data not shown). Similar correlations have been reported in non-IBC in clinical samples and cancer cell lines [44, 45, 49–51]. We did not find any correlation with histological ductal type and high grade, likely because of the relative small series size and the absence of grade 1 samples, but the percent of grade 3 was higher in the “PDL1-high” group (82% *vs* 70%), as observed in non-IBC.

The correlations between immune parameters and *PDL1* expression as binary variable - and as continuous variable (Wilcoxon's test; data not shown) - and the results of our supervised analysis showed that the microenvironment of “PDL1-high” IBC samples is different from that of “PDL1-low” samples and is suggestive of a strong local cytotoxic immune response. “PDL1-high” tumors showed a more dense T-cell infiltration – as already reported by other groups in non-IBC [43, 46, 51] with positive correlation between *PDL1* expression and the presence of elevated TILs-, higher expression of T-cell-specific and CD8+ T-cell-specific gene expression signatures, and higher expression of genes coding proteins related to the T-cell receptor. Furthermore, these tumors showed features of T-cell activation. Supervised analysis revealed overexpression of genes coding for T-cells differentiation factors and activation markers, cytotoxic effector molecules (granzymes, perforin, granulyzine), inflammation/anti-tumor cytokines such as interferon, and chemokines related to T-cells activation and homing. Molecules involved in the processing of endogenous antigens and presentation to immune cells and molecules involved in the degradation of cytosolic peptides were also overexpressed as well as markers of other cells of anti-tumor immunity (e.g. γδ-T-cells, NKG2D+ cells, dendritic-cells, B-cells). Finally, *PDL1* expression was associated with two expression signatures (LCK metagene [10] and 28-kinase metagene [11]) reflecting the T-cell cytotoxic immune response, and with a high probability of activation [53] of IFNα, IFNγ, and TNFα pathways. This pro-cytotoxic expression profile of “PDL1-high” samples was suggestive of an activated profile of differentiated T-cells (e.g. EOMES, CD27), T_H_1-biased (IL12 and IFN-induced pathways), and endowed with cytotoxic effector functions. However, we also noted that some T-cells infiltrating the tumor exhibited the phenotype of exhausted T-cells (CTLA4+/LAG3+/TIM3+/IDO+). T-cells exhaustion can be measured through the loss of secretion of IL2, IFNγ and TNFα, which occurs in a hierarchical manner. IFNγ and TNFα transcripts were still overexpressed in the “PDL1-high” group, suggesting that some activated T-cells might be progressively shifting toward a complete exhausted phenotype (still lacking the PD1+ CD160+ 2B4+ markers), most likely as a protective mechanism from the local inflammatory environment and the sustained IFNγ-mediated response. Reverting this exhausted phenotype through targeting of the surface receptors that inhibit T-cell function, such as PDL1 or LAG3, might considerably improve the local immune response and improve the patients' survival [57]. We hypothesize that *PDL1* expression (like the exhausted T-cell phenotype) represents a negative feedback mechanism that follows CD8+ infiltration [41, 58]. In non-IBC, *PDL1* mRNA expression is induced in the tumor microenvironment by activated TILs [43, 46] through the release of IFNγ [41, 47]. The correlations that we found between *PDL1* expression and the two signatures (LCK [10] and 28-kinase [11] metagenes) reflecting the cytotoxic T-cell response, and the activation [53] of IFNα, IFNγ, and TNFα pathways corroborate this hypothesis. Similarly, in a large series of breast cancers, a positive correlation was found between the expression of immunosuppressive checkpoint markers (PD-1, PDL1, CTLA4, and FOXP3) and the expression of proimmune markers, suggestive of a feedback activation of immunosuppressive pathways as part of the immune reaction [16]. Interferon gamma and other inflammatory cytokines, secreted by anti-tumor T_H_1-cells or macrophages, increase PDL1 expression, in response to immune-mediated attack [28], to decrease the cytotoxic local immune response.

These reactive mechanisms may explain the rather counterintuitive correlation between the high expression of *PDL1,* coding for an immunosuppressive molecule, and a better pathological response to neo-adjuvant chemotherapy. Within the 66 informative patients, the pCR rate was 50% in the “PDL1-high” group *versus* 22% in the “PDL1-low” group, with an OR equal to 3.4. Despite the relative small size of the series, *PDL1* expression had independent predictive value in multivariate analysis, when compared to other predictive features, including the classical histo-clinical variables and recent expression signatures identified in non-IBC. The correlation was also significant in uni- and multivariate analyses when *PDL1* expression was analyzed as continuous value (Wilcoxon's test; data not shown). Similar results were reported in non-IBC in two series of 265 [50] and 105 [51] samples, respectively. In fact, *PDL1* expression represents a surrogate marker of engaged CD8+ TILs, which are known to provide favorable predictive value for response to chemotherapy in non-IBC [59–61] and IBC [62]. Positive correlation of immunosuppressive markers with improved therapeutic response has been reported in other studies as well [9, 16, 63, 64].

Eighty-five stage 3 IBC cases were informative for MFS and OSS, but *PDL1* expression was not associated with survival. To date, only three breast cancer studies have studied the prognostic value of PDL1 expression in non-IBC [46, 49, 50], but results are rather contradictory. In a series of 398 cases studied by ISH assay *PDL1* mRNA expression was associated with longer recurrence-free survival [46] but protein expression) was associated with worse overall survival in a different series of 650 cases studied by IHC [49]. More recently, we reported the largest series ever analyzed of non-IBC (1, 080 patients for MFS and 3, 778 for OS) and showed that *PDL1* expression was not associated with survival in the whole series of samples, but was associated with better MFS and OS in the basal subtype. Our results in IBC agree with this recent study. Unfortunately, we could not apply prognostic analysis per subtype given the small number of IBC samples.

In conclusion, we show that *PDL1* is overexpressed in IBC as compared to non-IBC and that its overexpression as compared to normal breast is observed in 38% of IBC samples. In IBC, *PDL1* overexpression correlates with aggressive molecular subtypes (basal and ERBB2-enriched) and better pathological response to chemotherapy. This first report of *PDL1* expression in IBC shows clinical and biological relevance of *PDL1* expression in IBC, and independent predictive value for pathological response to chemotherapy in multivariate analysis. Its limitations include: retrospective nature and associated biases such as missing data, size of the series, absence of information for more patients with respect to response to chemotherapy and survival, and use of DNA microarrays that quantify mRNA expression level of both tumor and immune cells. Analysis of larger series, retrospective, then prospective is needed, as well as protein analysis when reliable antibodies are available. If confirmed, PDL1 expression might refine the prediction of pathological response in IBC and improve our ability to better tailor neo-adjuvant therapy. From a therapeutic point of view, because PDL1 expression in could indicate an adaptive mechanism of immune escape [65], the blockade of PDL1 should protect activated T-cells or reactivate inhibited T-cells and increase the anti-tumor immune response, thus improving the therapeutic response, notably when associated with immunogenic anticancer chemotherapy such as doxorubicin [66, 67]. Given the reported link between PDL1 expression and tumor response to PDL1-inhibitors [25, 26, 28–30], we suggest that IBC patients might be candidates to such new promising therapies. Clinical trials are urgently warranted.

## MATERIALS AND METHODS

### Breast cancer samples

Clinical samples were pre-treatment primary tumor samples from patients with invasive breast adenocarcinoma treated at the Institut Paoli-Calmettes (IPC, Marseille: 71 IBC and 139 non-IBC) and the General Hospital Sint-Augustinus (TCRU, Antwerp: 41 IBC and 55 non-IBC). Each patient gave written informed consent and the study was approved by our institutional review boards. IBC was defined according to the international consensus criteria [1]: rapid onset (less than 6 months) of breast erythema, edema, and/or “peau d'orange”, and/or warm breast, with or without an underlying palpable mass. IBC samples were diagnostic biopsies (AJCC stages 3–4) from consecutively treated patients, with available histo-clinical annotations and good-quality extracted tumor RNA. Non-IBC samples were surgical specimen in case of early stage disease (AJCC stages 1–2) and diagnostic biopsies in case of advanced stage disease (locally advanced: AJCC stage 3, and metastatic: AJCC stage 4). The final data set contained 306 samples, including 112 IBC samples and 194 non-IBC samples. We also profiled 4 normal breast samples that represented 1 pool of 4 samples from 4 healthy women (reduction mammoplasty), and 3 commercial pools of respectively 1, 2 and 4 normal breast RNA (Clontech, Palo Alto, CA).

Patients with IBC were treated with neo-adjuvant anthracycline-based chemotherapy often including taxane, and coupled with trastuzumab in more than 40% of ERBB2-positive cases. Chemotherapy was followed by mastectomy and axillary lymph node dissection for clinically non-progressive and consenting patients, then radiotherapy. The pathological response to chemotherapy was defined on the surgical specimens of both the primary tumor and the lymph nodes using Chevallier grading [68]. From the 112 IBC samples, 66 were available for pCR analysis. After radiotherapy, adjuvant hormone therapy was given to patients with ER-positive IBC, as well as adjuvant trastuzumab in ERBB2-positive cases. A total of 85 patients with non-metastatic IBC were assessable for survival analysis.

### Gene expression data analysis

Gene expression profiles had been generated in each institution using the same Affymetrix U133 Plus 2.0 human microarrays (Affymetrix^®^, Santa Clara, CA, USA) as previously described [69]. All data were MIAME-compliant and deposited in the Array-Express database (E-MTAB-1547 and E-MTAB-1006). Data analysis required pre-analytic processing. We first normalized each data set separately using Robust Multichip Average (RMA) [70]. Normalization was done in R using Bioconductor and associated packages. The gene annotation of hybridization probes was updated using NetAffx Annotation files (http://www.affymetrix.com; release from 01/12/2008). The probes were then mapped based on their EntrezGeneID. When multiple probes mapped to the same GeneID, we retained the one with the highest variance in a particular dataset. We then merged the two data sets by using COMBAT (empirical Bayes) [71] as batch effects removal method, included in the inSilicoMerging R/Bioconductor package [72]. The accuracy of normalization was controlled by principal component analysis (PCA) applied to the 306 tumors and the genes of PAM50 signature [73] (Supplementary Figure 1).

*PDL1* (CD274) expression was measured by analyzing the 227458_at Affymetrix probe set whose identity and specificity were verified using the NCBI program BLASTN 2.2.29+ and showed 100% accuracy. Expression in tumors (T) was measured as discrete value after comparison with mean expression in normal breast samples (NB): overexpression, thereafter designated “PDL1-high” was defined by a T/NB ratio ≥ 2 and no overexpression (“PDL1-low”) by a T/NB ratio <2. The cut-off, equal to 2, was arbitrarily chosen, but is frequently used with DNA microarray or qRT-PCR data, based on reproducibility experiments. Correlation analyses were also done with *PDL1* expression in continuous values. Thanks to the bimodal distribution of their respective mRNA expression and to avoid biases associated to immunohistochemistry (IHC) analyses across our two institutions, ER, PR, and ERBB2 expressions (negative/positive) were defined using mRNA expression data of *ESR1, PGR*, and *ERBB2* respectively as described [74]. The molecular subtypes of samples were defined using the PAM50 classifier [73] as previously described [75]. Because of the role of PDL1 in immunity, we also analyzed different immune multigene classifiers that better reflect the functional status of local immune response than assessment of TILs: the LCK metagene [10] and the 28-kinase metagene [11] - two prognostic gene expression signatures published in non-IBC-, three metagenes representing T-cells, CD8+ T-cells and B-cells [52], and three gene expression signatures of immune pathway activity: IFNα, IFNγ, and TNFα pathways [53]. Two gene expression signatures predictive for pathological response to neo-adjuvant chemotherapy in non-IBC were also applied: the FAC/T response signature [76] and the stromal signature [77].

Finally, to explore the biological pathways associated with *PDL1* expression in IBC, we applied supervised analysis to the 112 samples and the 14, 338 genes remaining after filtering (removal of probes with low and poorly measured expression and standard deviation inferior to 0.25 log_2_ units) for comparing the gene expression profiles between the “PDL1-high” *versus* “PDL1-low” tumors. We used Significant Analysis of Microarrays (SAM) [78] algorithm and considered *p*-values, corrected for multiple comparisons (false discovery rate; FDR), as significant when smaller than 0.05. Ontology analysis of the resulting gene list was based on the GO biological processes of the Database for Annotation, Visualisation and Integrated Discovery (DAVID; http://david.abcc.ncifcrf.gov/).

### Statistical analysis

Correlations between *PDL1* expression and histo-clinical factors were calculated with the Student's *t*-test for expression assessed as continuous variable and the Fisher's exact test for expression assessed as binary variable (PDL1-high and PDL1-low). The pathological complete response (pCR) to neo-adjuvant chemotherapy was defined as the absence of invasive cancer in both breast and axillary lymph nodes (Chevallier grades 1 and 2), whereas Chevallier grades 3 and 4 were considered as no-pCR. MFS was calculated from the date of diagnosis until the date of first distant relapse. OSS was calculated from the date of diagnosis until the date of IBC-related death. Follow-up was measured from the date of diagnosis to the date of last news for event-free patients. Survivals were calculated using the Kaplan-Meier method and curves were compared with the log-rank test. Univariate and multivariate survival analyses were done using a logistic regression analysis for pCR analysis (glm function and significance estimated by specifying a binomial family for model with a logit link) and Cox regression analysis for survival analysis (Wald test). Variables tested in univariate analyses included patients' age at time of diagnosis (≤50 years *vs* > 50), histological type (ductal *vs* other) and grade (3 *vs* 2; no grade 1 in our IBC series), ER, PR and ERBB2 status (positive *vs* negative), molecular subtypes, and *PDL1* expression status (“PDL1-high” *vs* “PDL1-low”). Variables with a *p*-value < 0.10 in univariate analysis were tested in multivariate analysis. All statistical tests were two-sided at the 5% level of significance. Statistical analysis was done using the survival package (version 2.30) in the R software (version 2.9.1; http://www.cran.r-project.org/). We followed the reporting REcommendations for tumor MARKer prognostic studies (REMARK criteria) [79].

## SUPPLEMENTARY FIGURES AND TABLES








