# Supplementary material for: PDL1 expression in inflammatory breast cancer is frequent and predicts for the pathological response to chemotherapy
Source: Oncotarget. 2015 Apr 11;6(15):13506–19. doi: 10.18632/oncotarget.3642 (PMC4537030; doi:10.18632/oncotarget.3642)
Supplement: Supplementary file 1 [file oncotarget-06-13506-s001.pdf]

SUPPLEMENTARY FIGURE AND TABLES

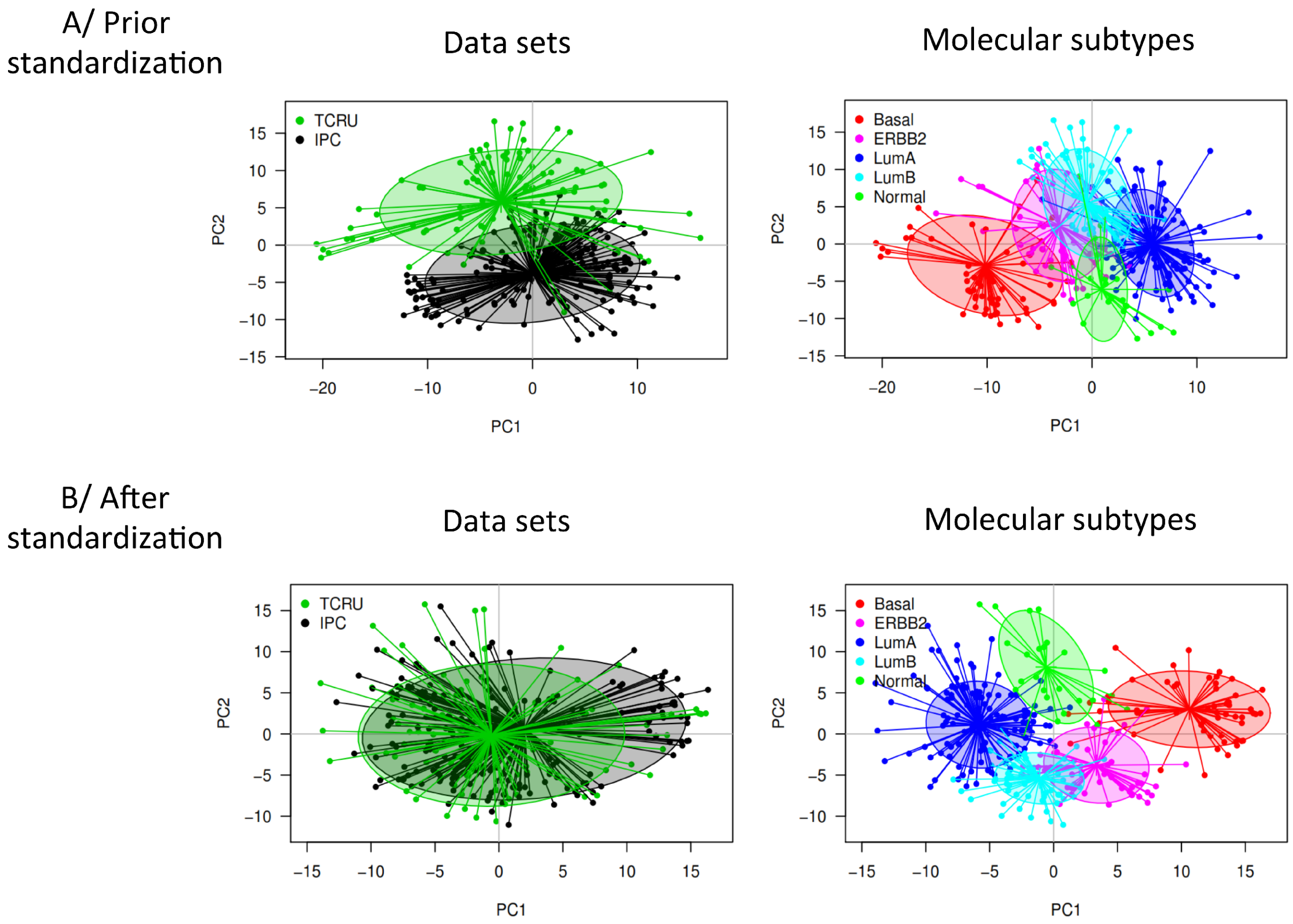

Supplementary Figure S1: Principal Component Analysis (PCA) of IBC and non-IBC samples and PAM50 genes before and after standardization. PCA was applied to the 306 breast cancer samples and the PAM50 genes. **A.** Before standardization, breast cancer samples in the 2D scatter plot representation are more grouped according to their origin data set (*left*: each color represents a set) than according to the molecular subtypes (*right*: dark blue for luminal A, light blue for luminal B, red for basal, pink for ERBB2-overexpressing, and green for normal-like). **B.** After standardization, samples are more clustered according to their molecular subtypes (*right*), rather than their origin data set is observed (*left*), clearly suggesting that the standardization has removed technical differences in gene expression while maintaining the information relevant to biological differences.

Supplementary Table S1: List of 1, 774 genes differentially expressed between IBC with *versus* without *PDL1* overexpression.

Supplementary Table S2: GO biological processes associated with the 1, 774 genes differentially expressed between IBC with *versus* without *PDL1* overexpression.

Supplementary Table S3: Univariate Cox regression analysis for MFS and OSS in patients with IBC.
